# The Transcription Factor Optomotor-Blind Antagonizes Drosophila Haltere Growth by Repressing Decapentaplegic and Hedgehog Targets

**DOI:** 10.1371/journal.pone.0121239

**Published:** 2015-03-20

**Authors:** Eléanor Simon, Isabel Guerrero

**Affiliations:** Centro de Biología Molecular Severo Ochoa, Consejo Superior de Investigaciones Científicas and Universidad Autónoma de Madrid, Madrid, Spain; University of Dayton, UNITED STATES

## Abstract

In Drosophila, *decapentaplegic*, which codes for a secreted signaling molecule, is activated by the Hedgehog signaling pathway at the anteroposterior compartment border of the two dorsal primordia; the wing and the haltere imaginal discs. In the wing disc, Decapentaplegic and Hedgehog signaling targets are implicated in cell proliferation and cell survival. However, most of their known targets in the wing disc are not expressed in the haltere disc due to their repression by the Hox gene *Ultrabithorax*. The T-box gene *optomotor-blind* escapes this repression in the haltere disc, and therefore is expressed in both the haltere and wing discs. Optomotor-blind is a major player during wing development and its function has been intensely investigated in this tissue, however, its role in haltere development has not been reported so far. Here we show that Optomotor-blind function in the haltere disc differs from that in the wing disc. Unlike its role in the wing, Optomotor-blind does not prevent apoptosis in the haltere but rather limits growth by repressing several Decapentaplegic and Hedgehog targets involved both in wing proliferation and in modulating the spread of morphogens similar to Ultrabithorax function but without disturbing Ultrabithorax expression.

## Introduction

Drosophila wings and halteres are homologous dorsal structures located in the second and third thoracic segments, respectively. In the larva, the primordia of wing and haltere are sacs of epithelial sheets called imaginal discs. Both structures differ in shape and size; however they share a common developmental program. Indeed, morphogens involved in developmental programs are expressed in a similar fashion in both discs. The acquisition of wing vs. haltere-specific features is controlled by the differential expression of the Hox gene, *Ultrabithorax* (*Ubx*), which is expressed in the haltere disc but absent in the wing disc [1, 2]. Ubx acts as a haltere identity selector, by impeding the activation of several downstream target genes which are part of signaling pathways common to both wing and haltere discs and by restricting the spread of morphogens within the disc [3–5].

*optomotor-blind* (*omb*, *bifid*—FlyBase) is one of the few Decapentaplegic (Dpp; a TGF-β family member) pathway targets [6] that is not repressed by Ubx in the haltere. Omb belongs to the Tbx protein family, which contains a characteristic DNA-binding domain [7], the T-box. Several developmental studies on this protein revealed that Omb regulates wing growth [6, 8–11] by preserving the wing disc from an excessive apoptosis [8, 9]. In the present study we report that, unlike its activities in wing development, Omb antagonizes haltere growth. Our data suggest that Omb controls haltere size by regulating Dpp and Hedgehog (Hh) signal dispersion and by repressing several morphogen targets implicated in wing growth. Importantly, Omb does not seem to regulate Ubx expression in the haltere, which suggests that some of the Dpp and Hh targets are repressed by both Omb and Ubx, either cooperatively or independently.

## Materials and Methods

### Drosophila strains

*dpp-Gal4* [12], *ptc-Gal4* [13], *ap-Gal4* [14], *Ubx-Gal4* ^*M1*^ [15], *col-Gal4* [16], *UAS-omb* [6], *tub-Gal80* ^*ts*^, *UAS-GFP*, *UAS-RFP* and *UAS-FLP* (Bloomington Stock Center), *omb* ^*282*^ [17], *Df(2L)32FP5* [18] (a deficiency that removed the genes *sal* and *salr*), *tkv* ^*a12*^ [19]. The *Df(2L)32FP5 tkv* ^*a12*^ chromosome was generated by meiotic recombination. *omb* ^*P1*^-*lacZ* [20], *5XQE-DsRed* [21] (a reporter of *vgQE*), *sal-lacZ* [22], *puc* ^*E69*^-*lacZ* [23], *iro* ^*rF209*^-*lacZ* [24], *dally-lacZ* [25], *brk* ^*X47*^-*lacZ* [26]. *Dp(3;3)P5* is a tandem duplication of the *bithorax complex* [27].

### Loss-of-function clones

*Df(2L)32FP5* mitotic null clones were induced in larvae *hs-FLP122*; *FRT40A Df(2L)32FP5*/*FRT40A UbiGFP*. *Df(2L)32FP5 tkv* ^*a12*^ mutant clones were induced in larvae *hs-FLP122*; *Df(2L)32FP5 tkv* ^*a12*^/*FRT40A UbiGFP*. *omb* ^*282*^ mitotic clones were induced in larvae *omb* ^*282*^ *FRT19A*/*hs-FLP122 hs-GFP FRT19A*. Clones were induced between 48 and 96 hours after egg laying by a 45 minute heat-shock, at 37°C. *omb* ^*282*^ mitotic clones were also induced in larvae *omb* ^*282*^ *FRT19A*/*FRT19A*; *UAS-FLP*/*ptc-Gal4 or omb* ^*282*^ *FRT19A*/*FRT19A*; *UAS-FLP*/*CyO*; *Ubx-Gal4* ^*M1*^/*TM6B* or *omb* ^*282*^ *FRT19A*/*FRT19A*; *UAS-FLP*/*CyO*; *Ubx-Gal4* ^*M1*^/*Dp(3;3)P5*. These clones were induced according to the spatio-temporal expression of the *ptc-Gal4* or the *Ubx-Gal4* ^*M1*^ driver.

### Transgene overexpression

Ectopic expression of *omb* was induced in the dorsal compartment of *ap-Gal4; UAS-omb*/*tubGal80* ^*ts*^ larvae by maintaining them at 17°C until early third instar (L3) before a shift to 29°C for 48h to inactivate the *tubGal80* ^*ts*^.

### Immunostaining of imaginal discs

The imaginal discs were stained following standard protocols using rabbit anti-β-gal (Jackson Laboratories), mouse anti-Wg, mouse anti-Ubx, mouse anti-Dlp (Aiowa Hybridoma Bank), rabbit anti-Caspase-3 (Cell Signaling Technology), rabbit anti-Doc2 [28] and with Phalloidin (PL)-TRITC (Sigma) to label the F-actin.

## Results and Discussion

### Sal impedes Dpp-mediated activation of *omb* in the proximal wing and haltere discs

**Fig 1:**
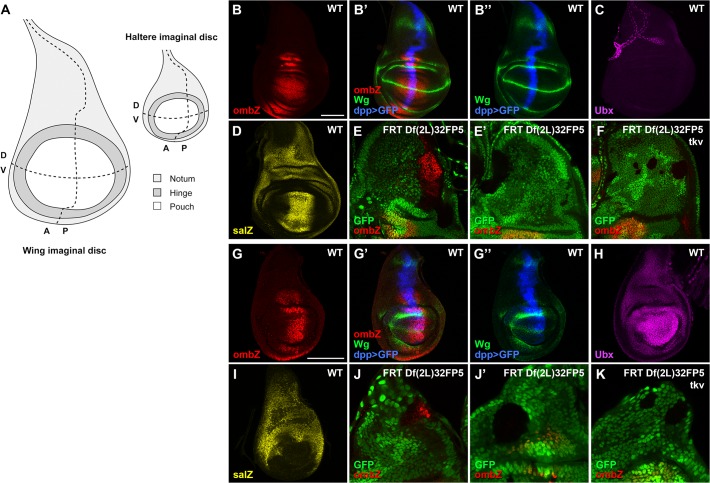
Regulation of *omb* in the proximal part of the wing and haltere discs. (**A**) Schematic representation of L3 wing and haltere discs. Dashed lines represent the disc compartment axes. A: anterior; P: posterior; D: dorsal; V: ventral. Proportions between both discs are respected. (**B**,**B’**,**B”**) Wild-type expression of *omb-lacZ*, *dpp-Gal4 UAS-GFP* (*dpp>GFP*) and Wg protein in the wing disc. (**C**) Wild-type expression of Ubx in the wing disc. Ubx protein is not expressed in the wing disc but marks tracheal cells. (**D**) Wild-type expression of *sal-lacZ* in the wing disc. (**E**,**E’**) *FRT Df(2L)32FP5* mutant clones located at the A/P compartment border (E) or far from it (E’) in the notum. *omb-lacZ* expression is only detected in (E). (**F**) A *FRT Df(2L)32FP5*, *tkv* double mutant clone located at the A/P border abolishes the *omb-lacZ* derepression observed in (E). (Note that this clone is smaller because *tkv* mutant cells fail to proliferate). (**G**,**G’**,**G”**) Wild-type expression of *omb-lacZ*, *dpp>GFP* and Wg in the haltere disc. (**H**) Wild-type expression of Ubx in the haltere disc. (**I**) Wild-type expression of *sal-lacZ* in the haltere disc. (**J**,**J’**) A *Df(2L)32FP5* mutant clone located at the A/P compartment border in the proximal haltere derepresses *omb-lacZ* expression (J) whereas one located far from the A/P axis has no effect on *omb-lacZ* expression (J’). (**K**) A *FRT Df(2L)32FP5 tkv* double mutant clone induced at the A/P boundary abrogates the *omb-lacZ* derepression observed in (J). Discs are oriented with posterior towards the right and dorsal upwards. Scale bars: 100μm.

In the wing imaginal disc (Fig. 1A shows wing disc organization), *omb* is expressed in a large domain around the anteroposterior (A/P) axis in the wing pouch and the presumptive hinge (Fig. 1B, B’). Both Dpp and Wingless (Wg) signaling pathways (see Fig. 1B’, B” for the ligands expression) activate *omb* expression in the wing pouch, whereas only Dpp signaling is required to activate its expression in the prospective hinge [6]. *dpp* is expressed along the whole A/P boundary in the wing disc; however, *omb* is not expressed in the proximal part of the disc, the notum (Fig. 1B’). This expression pattern presumes the presence of a repressor, which would prevent Dpp signaling to activate *omb* in these cells. The gene *spalt* (*sal*) is expressed in two broad domains in the wing disc (Fig. 1D)—one Dpp-dependent in the wing pouch, and another Dpp-independent in the notum [29]. As Sal can act as transcriptional repressor [30], we wondered whether it could be a repressor of *omb* expression in the notum. Indeed, loss-of-function clones of *sal* located in the notum relieve the repression (therefore inducing expression) of *omb* at the A/P compartment border (Fig. 1E), while clones located far from the A/P border do not induce *omb* expression (Fig. 1E’). Also, double mutant clones for *sal* and the type I receptor of the Dpp pathway, *thickvein* (*tkv*), do not shown an ectopic expression of *omb* (Fig. 1F), indicating that Dpp signaling is required to activate *omb* all along the A/P axis but its effect is masked in the proximal wing disc because of the presence of Sal. Note that in Fig. 1E, the derepression of *omb* within the *sal* mutant cells does not occurs within the whole clone, indicating that other factor(s) may be involved in *omb* repression in the notum.

In the haltere disc (see Fig. 1A for haltere disc organization), *omb* and *dpp* have a similar expression pattern than in the wing disc (compare Fig. 1B-B” to G-G”). The expression pattern of *wg* is slightly different between both structures; it is not expressed in the P compartment of the haltere disc (compare Fig. 1B’-B” to G’-G”) because of a repression by Ubx [3]. Also, Ubx protein impedes Dpp signaling to activate the distal expression of *sal* [3], which gets restricted to the proximal part (Fig. 1I). The wild-type expression pattern of Ubx is shown in Fig. 1C and 1H. As in the wing disc, mutant clones for *sal* in the haltere disc located close to the A/P compartment border derepress *omb* in the notum (Fig. 1J) while those induced far from the A/P axis do not (Fig. 1J’). Also, double mutant clones for *sal* and *tkv* in the proximal part of the haltere abolish the ectopic expression of *omb* (Fig. 1K). Altogether these results indicate that Sal impedes Dpp signaling to activate *omb* expression in the proximal part in both dorsal discs. This effect is independent of the Ubx input that each structure received differentially.

### Omb prevents haltere growth and does not inhibit JNK-mediated apoptosis in the haltere disc

**Fig 2:**
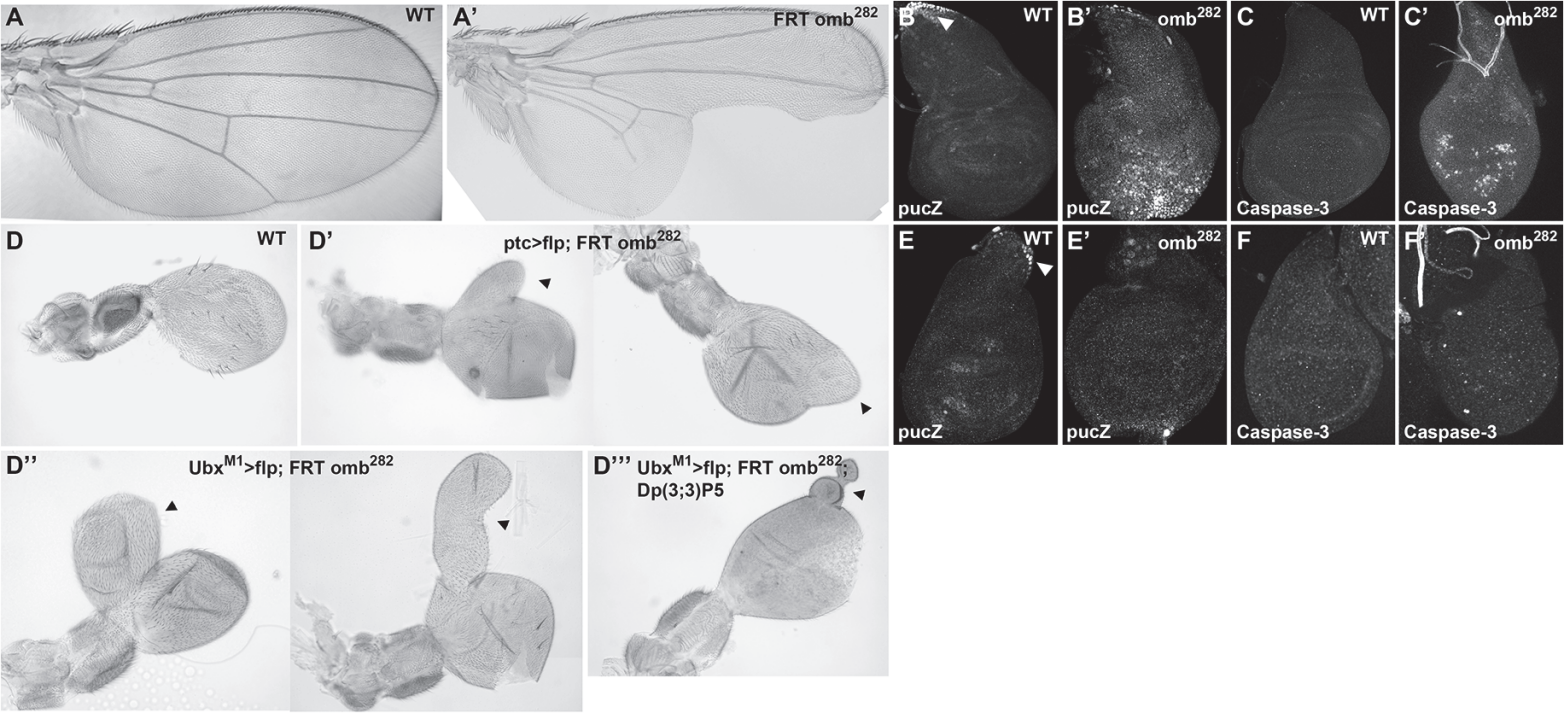
Comparison of wing and haltere phenotypes after removing *omb* function. (**A**,**A’**) Wild-type adult wing (A) and adult wing after induction of *omb* ^*282*^ mutant clones (A’) showing a missing epithelium in the central part. (**B**,**B’**) Wild-type L3 wing disc showing that *puc-lacZ* is only detected in the stalk (B; arrowhead), and *omb* ^*282*^ mutant wing disc showing ectopic activation of *puc-lacZ* in the central part of the disc (B’). (**C**,**C’**) Activated Caspase-3 is not detected in wild-type wing disc (C) but it is strongly activated in *omb* ^*282*^ mutant wing (C’). (**D**,**D’**,**D”**,**D”’**) Wild-type adult haltere (D); two representative examples of adult halteres showing overgrowths after the induction of *omb* ^*282*^ mutant clones under control of the *ptc-Gal4* driver (D’; arrowheads), or the *Ubx-Gal4* ^*M1*^ driver (D”; arrowheads). The large overgrowths observed in (D”) are smaller when *omb* ^*282*^ mutant clones are induced with the *Ubx-Gal4* ^*M1*^ driver in a bithorax complex duplication background (D”’; arrowhead). (**E**,**E’**) *puc-lacZ* is neither detected in wild-type haltere disc, except in the stalk (E; arrowhead), nor in *omb* ^*282*^ mutant haltere (E’). (**F**,**F’**) Activated Caspase-3 is neither detected in wild-type haltere disc (F), nor in *omb* ^*282*^ mutant haltere (F’). Scale bars: 250μm.

Strong loss-of-function *omb* alleles are lethal at the pupal stage [6]. Thus, to study adult phenotypes, we removed *omb* function in mitotic recombinant clones. Wings containing big *omb* mutant clones lack the central region (compare Fig. 2A and A’) [6]. This phenotype is due to Jun N-terminal protein kinase (JNK)-mediated apoptosis [8]. Indeed, *puckered* (*puc*)-*lacZ* expression, a reporter for the JNK pathway, is activated in *omb* mutant wing discs (compare Fig. 2B and B’) [8], and the active form of Caspase-3, a component of the apoptotic pathway, is detected in those discs (compare Fig. 2C and C’) [8].

To test whether Omb has a similar role in the haltere, we first analyzed the adult haltere phenotype after eliminating *omb* function in mutant clones. Because of the relative small size of the haltere, we increased the probability of getting mutant cells in the *omb* expressing territory by driving the *FLP* expression in the *ptc-Gal4* domain, which is expressed in a stripe of cells at the A/P compartment border. Halteres with such clones do not show lack of tissue. Rather, they present haltere-like tissue overgrowths (compare Fig. 2D and D’; arrowheads) in 17% of the flies observed (n>250). We also induced *omb* mutant clones by expressing *FLP* under the control of the *Ubx-Gal4* ^*M1*^ driver. Under this condition, 23% of the flies observed (n>300) have overgrowths in the haltere, which can reach the size of a normal haltere (Fig. 2D”; arrowheads). *Ubx-Gal4* ^*M1*^ flies carry a mutation in *Ubx* [15] and could also contribute to these overgrowths. To test this hypothesis, we induced *omb* clones in a background containing a duplication for *Ubx*. In this case, the overgrowths were smaller but still observed (Fig. 2D”’; arrowhead). This result indicates that *omb* mutant cells respond differentially depending on Ubx levels.

We then analyzed the effect of Omb on the apoptotic pathway in the haltere disc. Almost no activation of the JNK pathway was detected in *omb* mutant haltere discs (compare Fig. 2E and E’). Consistently, no significant apoptosis, detected by the activation of Caspase-3, was induced in those *omb* mutant discs (compare Fig. 2F and F’). Overall, these results reveal a divergent Omb function between the wing and the haltere: contrary to the wing, Omb represses tissue growth in the haltere and does not inhibit JNK-mediated apoptosis in the haltere disc.

### In the haltere disc, Omb represses various genes involved in wing growth

**Fig 3:**
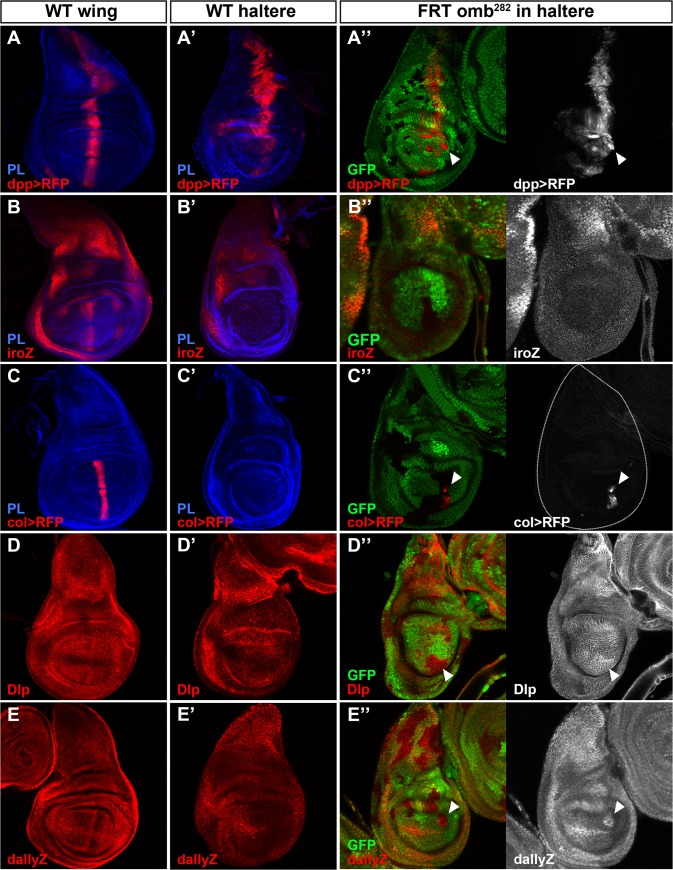
Regulation of Hh signaling targets by Omb in the haltere disc. (**A**-**E**) Wild-type expression of *dpp>RFP* (A), *iro-lacZ* (B), *col>RFP* (C), Dlp (D) and *dally-lacZ* (E) in wing discs. (**A’**-**E’**) Wild-type expression of *dpp>RFP* (A’), *iro-lacZ* (B’), *col>RFP* (C’), Dlp (D’) and *dally-lacZ* (E’) in haltere discs. (**A”**-**E”**) *omb* ^*282*^ mutant clones induced in the haltere discs showing no change in *iro-lacZ* (B”) expression, an upregulation of *dpp>RFP* (A”), Dlp (D”) and *dally-lacZ* (E”) expression (arrowheads) and an ectopic expression of *col>RFP* (C”; arrowhead). In all cases, 100% of the clones observed induce in the Omb expression domain show alterations of the markers previously mentioned (n≥12), however *col>RFP* is only observed in 50% of these clones and as illustrated in (C”) only in a part of the clone (n = 12). Clones are marked by the lack of GFP.

We hypothesized that the overgrowths of cells in the haltere triggered by the removal of *omb* function could be due to alteration of the downstream targets of the signaling pathways normally involved in wing growth, and known to be repressed by Ubx in the haltere [3, 31]. Hh signaling is involved in wing growth via the activation, among other targets, of *dpp* (Fig. 3A), the *iroquois* (*iro*) *complex* genes (Fig. 3B) and the transcription factor *collier*/*knot* (col; Fig. 3C) [24, 32, 33]. In the haltere disc, *dpp* is expressed in a similar domain than in the wing disc but at lower level (Fig. 3A’), while *iro* and *col* expression (Fig. 3B’, C’) is missing in the haltere pouch disc because of a downregulation or a repression by Ubx respectively [4, 34, 35]. In *omb* mutant clones, we observed an upregulation of *dpp* expression (Fig. 3A”; arrowhead). *iro* expression was not induced in similar clones (Fig. 3B”). However, a derepression of *col* expression in *omb* mutant cells located close to the A/P compartment border was found (Fig. 3C”; arrowhead), coinciding with the homologous region where *col* expression is induced by Hh in the wing disc.

It has been also reported that Ubx modulates morphogen signaling in the haltere through transcriptional regulation of the two glypicans Division abnormally delayed (Dally) and Dally-like (Dlp) [4, 5, 31, 36]. Dlp and Dally play cooperative and distinct roles in modulating Hh and Dpp gradient and signaling [37]. Both proteins are present in the haltere disc but at a low level in comparison with their expression level in the wing disc (compare Fig. 3D, E to D’, E’ respectively) due to Ubx in the haltere [4, 5, 36]. In support of the new role of Omb in the haltere, both glypicans were upregulated in *omb* mutant clones induced in the haltere disc (Fig. 3D”, E”; arrowheads).

**Fig 4:**
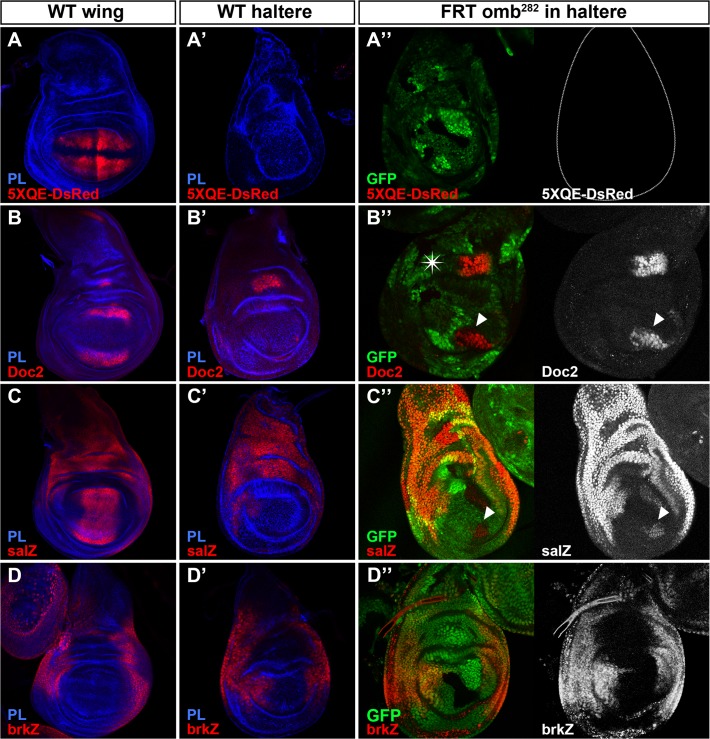
Regulation of Dpp signaling targets by Omb in the haltere disc. (**A**-**D**) Wild-type expression of *5XQE-DsRed* (A), Doc2 (B), *sal-lacZ* (C) and *brk-lacZ* (D) in wing discs. (**A’**-**D’**) Wild-type expression of *5XQE-DsRed* (A’), Doc2 (B’), *sal-lacZ* (C’) and *brk-lacZ* (D’) in haltere discs. (**A”**-**D”**) *omb* ^*282*^ mutant clones induced in the haltere discs showing no change in *5XQE-DsRed* (A”) nor in *brk-lacZ* (D”) expression. In similar clones, Doc2 is fully derepressed (B”), while *sal-lacZ* is partially derepressed (C”). In all cases, 100% of the clones induced in the Omb expression domain show alterations of the markers previously mentioned (n≥12). Clones are marked by the lack of GFP. The star in (B”) marks the normal expression pattern of Doc2 in the haltere hinge.

In the wing pouch, in addition to *omb*, Dpp pathway activates *vestigial* (*vg*)-*QE* (Fig. 4A; visualized with the *5XQE-DsRed* reporter), which is known to be involved in wing growth and sufficient to promote wing identity [38], the T-box *Dorsocross* (*Doc*)*2* (Fig. 4B) [39], which is involved in proximal wing pouch growth [40] and *sal* (Fig. 4C), which stimulate cell proliferation [41]. None of these Dpp signaling targets is expressed in a wild-type haltere pouch (Fig. 4A’, B’ and C’). Despite the fact that, *vg-QE* expression is not induced in *omb* mutant clones in the haltere pouch (Fig. 4A”), we were able to detect ectopic expression of *Doc2* and *sal* in similar clones located close to the A/P compartment border in a region homologous to their normal expression domains in the wing (Fig. 4B” and 4C” respectively; arrowheads). The level of derepression of Doc2 protein in those clones appears to reach its endogenous level of expression in the wing disc, suggesting a full derepression. However, the level of expression of *sal* in *omb* mutant clones was weaker than its endogenous level in the wing disc. We wondered whether this latter effect could be mediated by the misexpression of *brinker* (*brk*), which code for a general repressor of Dpp-dependent genes [42–44], expressed normally in the lateral part of both wing and haltere discs (Fig. 4D, D’ respectively). However, no appreciable change in *brk* expression was observed (Fig. 4D”), indicating that another factor would impede a full derepression of *sal*. Overall, these results suggest that the haltere overgrowths observed upon removal of *omb* function could be promoted by the derepression of *col*, *Doc2* and *sal*, genes involved in wing proliferation, and by *dally* and *dlp* which are implicated in the modulation of morphogen dispersion and activity. On the contrary, the lack of derepression of *vg* and *iro*, both required for wing identity, is consistent with the fact that the adult halteres, where *omb* mutant clones have been induced, do not present a transformation to wing tissue or a vein pattern.

### Omb does not regulate Ubx expression levels

**Fig 5:**
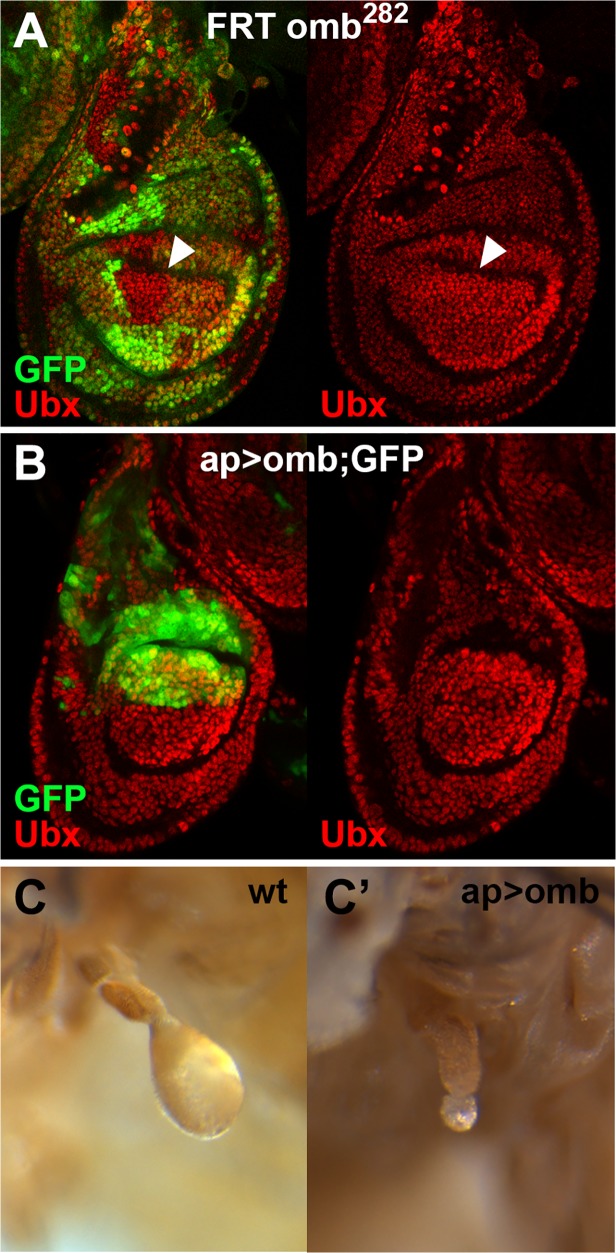
Loss- or gain-of- function of *omb* does not affect Ubx expression in the haltere disc. (**A**) Level of Ubx expression is not modified in *omb* ^*282*^ mutant clones in the haltere disc (arrowhead). (**B**) Level of Ubx expression is neither modified when *omb* is expressed ectopically in the dorsal compartment using the *ap-Gal4; UAS-GFP* driver. (**C,C’**) Wild-type adult haltere (C) and an example of adult haltere phenotype with the same genotype as in (B), showing haltere atrophy (C’).

As Ubx represses several Hh and Dpp targets in the haltere [3, 31], we wondered whether the derepression of the targets observed in *omb* mutant clones could be mediated by an alteration of Ubx expression. No change of the level of Ubx protein expression was detected in *omb* mutant cells (Fig. 5A; arrowhead). Conversely, the ectopic expression of *omb* in the dorsal compartment of the haltere disc is not sufficient to affect Ubx expression (Fig. 5B). Interestingly, we note that those discs present a reduction of the dorsal compartment compared to wild-type discs (Fig. 5B and not shown). In agreement with this observation, most of the adult flies present a reduction of haltere size and some are even devoid of the appendage (compare Fig. 5C to 5C’ and not shown); this represents the opposite phenotype of the overgrowths observed in *omb* mutant clones in adult halteres (Fig. 2D’, D”).

## Conclusions

Our results indicate that Omb restricts haltere growth by repressing the activity of Dpp and Hh signaling which is probably coupled with a control of ligands spreading within the disc. In addition, these effects do not seem to be mediated by changes in Ubx expression, however it is likely that Ubx limits the response of the removal of *omb*, which could explain why *sal* is only partially derepressed in those cells. Further experiments will be needed to understand, at a molecular level, how Omb represses Ubx targets in the haltere. It is known that Hox proteins do not act alone but bind cofactors, which confer them specificity in the DNA binding and in gene expression regulation [45]. Therefore, it is possible that Ubx requires Omb as a cofactor to specifically repress a subset of Hh and Dpp targets. Alternatively, Ubx and Omb may act in parallel in this repressive process.
